# Reliability of MyotonPro in measuring the biomechanical properties of the quadriceps femoris muscle in people with different levels and types of motor preparation

**DOI:** 10.3389/fspor.2024.1453730

**Published:** 2024-08-29

**Authors:** Robert Trybulski, Adrian Kużdżał, Michał Wilk, Jakub Więckowski, Krzysztof Fostiak, Jarosław Muracki

**Affiliations:** ^1^ Medical Center Provita Żory, Żory, Poland; ^2^ Medical Department, Wojciech Korfanty Upper Silesian Academy, Katowice, Poland; ^3^ Institute of Health Sciences, College of Medical Sciences, University of Rzeszów, Rzeszów, Poland; ^4^ Institute of Sports Science, The Jerzy Kukuczka Academy of Physical Education, Katowice, Poland; ^5^ National Institute of Telecommunications, Warsaw, Poland; ^6^ Gdansk University of Physical Education and Sport, Gdansk, Poland; ^7^ Institute of Physical Culture Sciences, Department of Physical Culture and Health, University of Szczecin, Szczecin, Poland

**Keywords:** repeatability, muscle tension, stiffness, elasticity, sports medicine

## Abstract

The aim of this research was to evaluate the reliability of the measurements of biomechanical parameters of the muscles of athletes representing different disciplines as well as untrained people. Ninety-four young, healthy male individuals participated in the study and were divided into five subgroups: footballers (*n* = 25), volleyballers (*n* = 14), handballers (*n *= 19), MMA fighters (*n* = 16), and undrained group (*n* = 20). All of the participants underwent measurements of stiffness (S), muscle tone (T) and elasticity (E) by two independent measurers using MyotonPro equipment. Analysis was conducted on two different parts of the quadriceps femoris: rectus femoris (RF) and vastus medialis (VM. Consequently, the comprehensive analysis comprised 564 measurements (94 participants * 3 parameters = 282 * 2 measurers = 564). The results proves high reliability of the myotonometry (Pearson's CC over 0.8208–0.8871 for different parameters, ICC from to 0.74 to 0.99 for different muscles and parameters) excluding only stiffness for the VM which was characterized withlow ICC of 0.08 and relatively highest between the examined parameters MAE% of 8.7% which still remains low value. The most significant differences between the parameters in examined groups were observed between MMA fighters and volleyballers in terms of muscle tone and elasticity of the VM (correlation of 0.14842 and 0.15083 respecitively). These results confirm the usability of myotonometry in measuring the biomechanical properties of the muscles in different sports groups and confirm the independence of the results obtained from the person performing the measurement.

## 1 Introduction

Understanding the biomechanical properties of muscles is not just a matter of scientific interest. It has direct and significant implications in medicine and athletic training (1), quality of life (2), injury prevention (3), athletic achievements (4), muscle recovery (5), and even lifespan (6).

Many methods are available in the scientific literature for assessing the biomechanical properties of myofascial tissue, broadly divided into invasive and non-invasive, subjective and objective (7). The following methods can be used to assess the biomechanical properties of tissue: shear wave elastography (SWE), magnetic resonance imaging (MRI), called magnetic resonance elastography (MRE), Doppler laser vibrometer, shear wave propagation accelerometer (8). Tissue deformation and shear wave excitation can also be performed mechanically, and one such device is a myotonometer (9). Its advantages are relatively simple operation, affordable price, ability to perform measurements in variable conditions, and ease of measurement (10). Despite the general availability of various tools for measuring the biomechanical properties of myofascial tissue (11), it is necessary to clarify what is the repeatability of measurements for various measuring devices (12), measurement places (1), individual variability (13), type of sport practiced (14), measurement position (4).

Previous studies assessed the repeatability of the measurement by Myoton depending on the number of head depressions (15), the amount of force generated by the muscles (16), age (17) and gender (18), stretching muscle (19), between different muscles (20) comparison within a given sport (21–23) and between two different disciplines (24). There is abundant scientific evidence confirming the repeatability of measurements for the same point at specific time intervals (17, 25), measurements using various researchers (16), comparing different measurement tools (26–28), comparing *in vivo* with *in situ* studies (12, 29) assessing changes in diseases of the musculoskeletal system (7, 30, 31) assessing changes in various regenerative methods (5, 32) post-exercise changes (5, 33) (ref), changes depending on the position (20, 34), body flow resistance (BFR) (35).

Elasticity is a mechanical property of soft tissues characterized by the ability to return to their original shape after a deforming force, depending mainly on the architecture of collagen fibers (36). Muscle tone, often referred to as the “most neurogenic property” (37), highlights the complex role of the nervous system in coordinating and regulating muscle activity to achieve efficient and flexible movement patterns (38). Stiffness, or the resistance of a material or structure to deformation (3), has been found to influence the risk of injury (39). Stiffness is influenced by many variables, including the time of rise of strain energy and its type. These observations regarding pain and stretch speed further shed light on the multifaceted nature of muscle biomechanics (40). The difficulty in establishing normative values that could be used as a standard in sports medicine and beyond prevents researchers and clinicians from determining whether myotonometry is an accurate monitoring method capable of quantifying post-intervention biomechanical properties and inter-individual variability (41). Previous research has shown that men exhibit significantly greater muscle stiffness than women (42), and the difference in stiffness varies depending on the level of muscle contraction (43, 44). Still, no current information is available to determine whether and how stiffness differs between the sexes in posture. This information is necessary to decide whether myotonometry can be used as a clinical tool to measure muscle biomechanical properties (15).

In these three parameters, possible differences are expected between the groups training different sports and in comparison with untrained people because of different movement characteristics, proportions of type of muscle work (i.e., isometric work is typically often used in MMA training), training routines, and varied loads during competition in these sport disciplines (football, handball, volleyball, MMA, and untrained group).

Knowledge of the factors that may influence the biomechanical variables measured by MyotonPro seems crucial in assessing the possibility of increasing the reliability of measurement systems (1). Although myotonometry in the scientific literature has demonstrated moderate to excellent test-retest reliability (ICC = 0.60–0.98) in measuring muscle biomechanical properties (45) a gap remains in the small number of studies assessing reliability and precision [standard error measurements (SEM) and minimum detectable change (MDC)] (46).

Despite the widespread use of MyotonPro (9, 10), there is still a gap in the scientific literature assessing the repeatability and reliability of *in vivo* MyotonPro testing between different groups of athletes. Still, there is a lack of knowledge if the site of measurement, particular muscle, level of motor preparation, or sports discipline can influence the reliability of the measurements.

Our study aimed to determine the usefulness and repeatability of MyotonPro in measuring the stiffness, elasticity, and tension of two sites on the quadriceps femoris muscle in people with different levels of motor preparation, thus providing new insight into the biomechanical properties of myofascial tissue depending on the type of muscle loads. As part of our research innovation, we examined the repeatability of measurements in a broad group of people with different levels of motor preparation, from elite volleyball players to random people who do not practice any sports discipline. Considering such diverse groups and two measuring people, the attempt to assess the repeatability of MyotonPro measurements and the characterization of the biomechanical properties of muscles is innovative.

## 2 Methods

### 2.1 Study design

**Figure 1:**
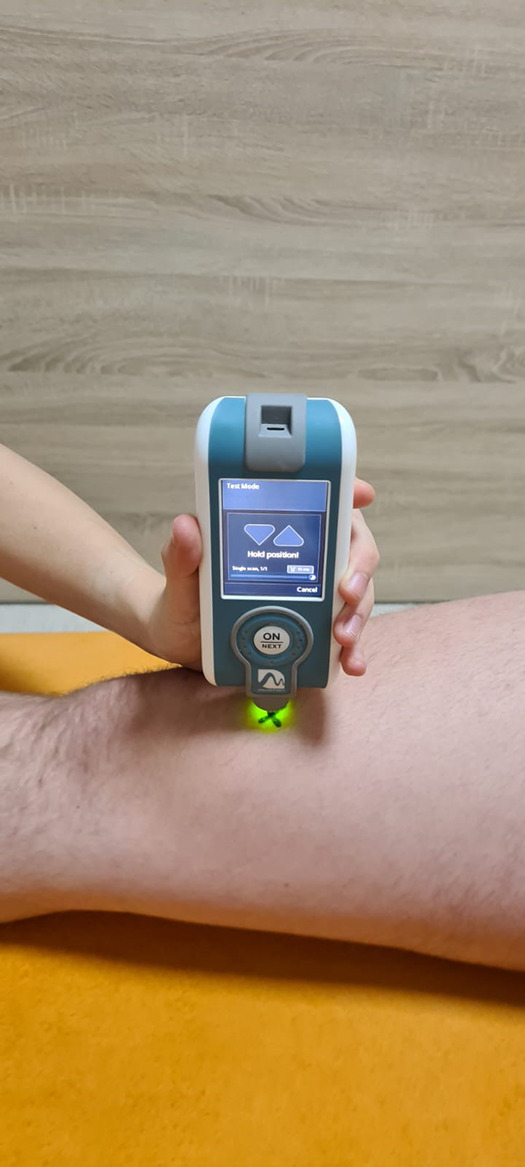
Myotonpro measurement on VM.

**Figure 2:**
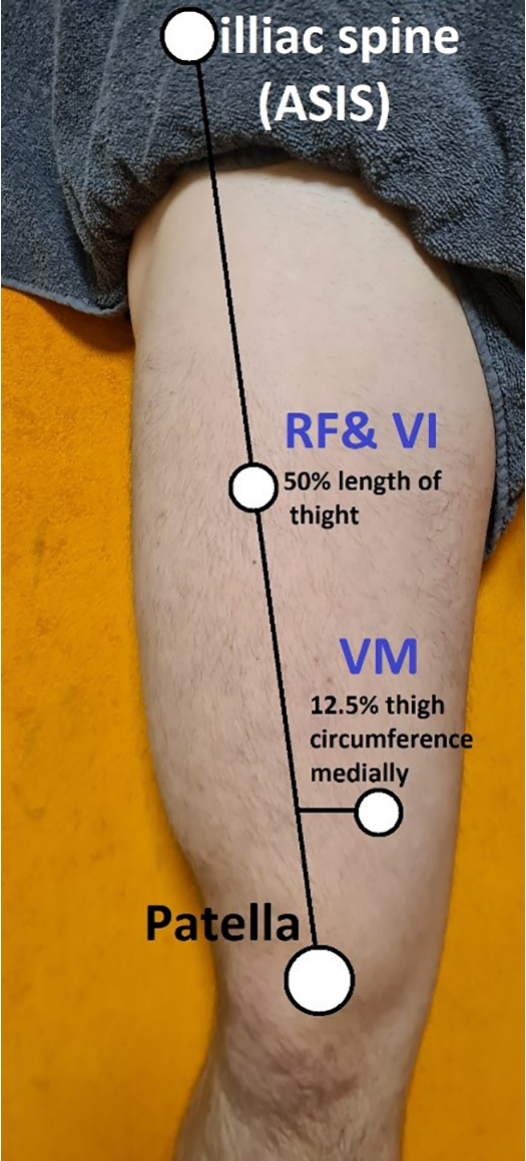
Assessment points on the quadriceps muscle.

The study was conducted with two blinded investigators who assessed muscle tone, elasticity, and stiffness using MyotonPro (Myoton AS., Tallinn, Estonia) on the two points—Rectus Femoris (RF) and Vastus Medialis (VM) of the quadriceps muscle of the dominant leg. The assessments were conducted at the Provita Medical Center (Żory, Poland) in standardized positions (Figure 1). After identifying and marking the broadest cross-sectional area of the RF and VM (Figure 2) (34), the following measurements were performed in all participants: muscle tension [T—(Hz)], stiffness [S—(N/m)], and elasticity [E—(arb—relative arbitrary unit)]. First, one researcher assessed both heads of the muscle (RF and VM), and after a 30 s break, the second researcher took an assessment. Those performing the assessment did not have access to the recorded data entered by the third researcher—a student. Before the assessment participants had 24 h rest without any training or excessive fatigue. The exact temperatures and air humidity prevailed in the measurement room. Tests were performed between 10 a.m. and 12 a.m. The study was approved by the ethical committee of the National Council of Physiotherapists (no. 9/22 of April 6, 2022) and registered in the clinical trials register at doi: 10.1186/ISRCTN90040217. The study was conducted in accordance with the Declaration of Helsinki. The Report Reliability and Compliance Research Guidelines were followed (47).

### 2.2 Participants

The study enrolled ninety-four male volunteers (*n* = 94) divided into five groups depending on the motor preparation level and the sport type practiced (Table 1). A group of elite volleyballers (*n* = 14) group of amateur footballers (*n* = 25), a group of amateur MMA (mixed martial arts) fighters (*n* = 16), a group of professional handballers (*n* = 19), and non-training volunteers—general population (*n* = 20). General inclusion criteria included age 18–40, a minimum of three years of experience training a given discipline, and training at least three times a week (not applicable for the untrained group), no injury at the time of the investigation, no injuries past 3 months before the start of the study, no history of injuries in the knees, thighs or hips area and overall good health status. Based on McKay's participant classification scheme, individual volunteers were Tier 2, 3, and 4: Highly Trained/National Level (48). Study exclusions included elevated pre-test blood pressure (>140/90 mm Hg), currently treated injuries, damaged skin, or unspecified skin lesions at the measurement sites, and the prohibition against taking painkillers or medications that change muscle tension. Exclusions were also made in the event of extreme fatigue, fever, infection, or at the explicit request of the participant (49). Written informed consent was obtained from participants after reviewing the study conditions. Before the tests, participants were required to refrain from training for 24 h. Exclusion from the study could occur at any time during the study at the participant's request. The authors declare that they have the consent of the participant in the photo to use the image in a scientific article.

**Table 1:** Comparative analysis of physical characteristics of the five examined groups, results are presented as mean ± SD.

Group	Footballers (*n* = 25)	Volleyballers (*n* = 14)	Handballers (*n* = 19)	MMA fighters (*n* = 16)	Untrained (*n* = 20)
Age [years]	21.8 ± 3.6	28.2 ± 4.2	25.2 ± 5.7	29.4 ± 5.5	26.7 ± 7.5
Tr. Experience [years]	13.4 ± 3.1	16.8 ± 4.8	13.4 ± 6.0	8.9 ± 4.8	0.0 ± 0.0
Body mass [kg]	76.7 ± 8.2	92.4 ± 10.6	92.1 ± 11.5	84.8 ± 13.7	78.0 ± 10.1
Body height [m]	1.8 ± 0.05	1.96 ± 0.10	1.86 ± 0.05	1.78 ± 0.05	1.81 ± 0.05
BMI [kg/m^2^]	23.5 ± 1.7	24.0 ± 1.1	26.1 ± 2.8	26.6 ± 4.9	23.6 ± 2.4

BMI, body mass index.

### 2.3 Measurements—myotometry

Measurements were made using a myotonometer (MyotonPRO, Estonia 2021) (Figure 1). All the measurements were made in the Provita Żory Medical Center on Saturdays between 10 and 12 a.m. The measurements were all in the same room with a controlled temperature inside of 22℃ and a relative humidity of 50%. To determine the measurement points, a line was drawn between the anterior superior iliac spine (ASIS) and the medial corner of the patella. The measurement point on the rectus femoris (RF) was designated on halfway along this line. The measurement point on the vastus medialis (VM) was localized on the widest part of the medial head of the quadriceps femoris and 12.5% of the thigh circumference medially. The measurements were performed on the dominant leg. The measurements were recorded in accordance with the methodology provided by the producer of the equipment. The measurement points are presented on Figure 2. The study was conducted in the off-season period, and every participant had 24 h of rest or physical exercise before the measurements. After marking the place with a marker, the measurement was made by immersing the myotonPro probe head three times in a specific place in a standardized position, lying on the back with a 20 cm diameter roller under the knees (32). The probe had to be placed perpendicular to the measured surface, and in case of deviations, the device automatically recorded the measurement error and ordered the measurement to be repeated (Figure 2).

Myoton is a digital device with a body and a depth probe (Ø 3 mm). An initial pressure (0.18 N) is applied to the surface through the probe, which exerts a dynamic shear force on the underlying material. The device then releases a mechanical pulse (0.4 N, 15 ms), quickly deforming the material. Myotonometry is considered as reliable measurement method and can detect differences in the physical properties of muscle fibers (10, 28). The measurement method involves recording the damped natural vibrations of soft biological tissue in the form of an acceleration signal and then simultaneously calculating the parameters of the stress state and biomechanical properties, such as muscle tension (T), dynamic stiffness (S), and elasticity (E) (10). Units of measurement are calculated based on logarithmic formulas (34). In the analysis of measurements for each researcher, a single application of the probe to the tissue at the measurement site was used, with the probe deforming it three times.

Several potential variables may influence the viscoelastic properties of tissues measured using MyotonPro. One of them is thixotropy, i.e., the property of some materials (including biological ones) to change viscosity under the influence of mechanical action, for example, movement or vibration. In the context of muscles and connective tissue, thixotropy refers to the reduction in viscosity (i.e., “loosening”) of tissues under exercise, physical activity, or diet. An example of thixotropy in biological tissues is that after warming up or being exposed to high temperatures, muscles, and joints become more flexible and less stiff [Schleip R. Fascial plasticity—A new neurobiological explanation Part 2. *J. Bodyw. Mov. Ther*. 2003; 7:104–116. doi: 10.1016/S1360-8592(02)00076-1].

### 2.4 Statistical methods

The statistical analysis of the measured by MiotonPro variables was structured to accommodate three distinct scenarios: individual assessments for each muscle (RF, VM) and joint analyses encompassing integrated measurements from analyzed muscles (Both). These scenarios allowed for exploring measurement correlations under varying conditions, providing insights into the consistency and reliability of the evaluation process across different contexts.

By evaluating the correlation between data obtained from these measurements, the research aimed to indicate whether variations in measurement specialists might lead to significant discrepancies in the received data. Four key metrics were employed to achieve this objective: the Pearson correlation coefficient (50), mean absolute error (MAE) (51), mean squared error (MSE) (52), and intraclass correlation (ICC) (53).

These measures were justified based on their capabilities in evaluating different aspects of agreement between measurements. The Pearson correlation coefficient served as a fundamental tool for quantifying the linear relationship between the data obtained by the two specialists. Its utilization was motivated by its ability to provide a straightforward indication of the degree of agreement or divergence between the measurements. The correlation values placed in range [−1, 1] can clearly indicate how strong correlation value is, with greater values showing higher correlation of analyzed data. Meanwhile, the inclusion of mean absolute error and mean squared error offered complementary perspectives on the consistency of measurements. MAE provided a straightforward assessment of the average magnitude of differences between measurements, offering insights into the typical level of agreement. MSE, with its emphasis on larger discrepancies, provided a more sensitive evaluation of agreement by highlighting significant variations between measurements. The ICC metric was included to assess the agreement between different groups in the study regarding the measurements taken by two distinct specialists. ICC quantifies the consistency or agreement among ratings provided by different groups for each variable. It provides insights into the reliability of measurements across various groups, with values closer to 1 indicating higher agreement. These metrics were chosen to provide insights into the magnitude and distribution of discrepancies, thereby enhancing the comprehensiveness of the analysis.

## 3 Results

### 3.1 Group characteristics

The gathered data compare demographic and physical attributes among five groups: Footballers, Volleyballers, Handballers, MMA fighters, and the young healthy untrained males. Each group's characteristics regarding age, training experience, weight, height, and body mass index (BMI) are presented in Table 1. This table shows the diverse physical profiles of individuals across different sports.

### 3.2 Muscle properities results

For both RF and VM muscles, the Pearson correlation coefficient values were relatively high across all aspects analyzed (Tension, Elasticity, and Stiffness). Specifically, for RF, the highest correlation is observed in Stiffness (0.8876), followed closely by Elasticity (0.8298) and Tension (0.8164). For VM, Stiffness exhibits the lowest correlation (0.7253), followed by Tension (0.8298) and Elasticity (0.8369). When measurements from both muscles are combined, the correlations remain consistently high across all aspects, with the highest correlation observed in Elasticity (0.8871). It should be noted that the Pearson correlation coefficient values obtained from the analysis indicated a strong correlation between the measurements performed by the two examined specialists.

For RF, the lowest MAE is observed in Stiffness (0.0992), followed by Muscle Tone (0.5191) and Elasticity (9.9148). For VM, the lowest MAE was also observed in Stiffness (0.1341), followed by Muscle Tone (0.9638) and Elasticity (23.1064). When measurements from both muscles are combined, the MAE values show a similar trend, with the lowest MAE observed in Stiffness (0.1167). A significant difference between the obtained values is observed for the Elasticity. This is due to the difference in the base magnitudes in which the values for this parameter are expressed. Thus, it was meaningful to analyze the relative measure to compare better the obtained MAE values. For this purpose, they were transformed into percentage values based on the mean values calculated for the given muscle and given measurement aspect. This analysis showed that the lowest percentage value was noted for the RF muscle and tension aspect, which equaled 3.4%. The highest percentage value was for VM and Stiffness (8.7%), while a slightly lower value was observed for the VM and Elasticity (8.5%). It shows that for the VM measurements, the differences between measurers were visibly more extraordinary than in the case of the RF muscle.

Mean Squared Error (MSE) further evaluates the accuracy of measurements by quantifying the average squared differences between actual and predicted values. Lower MSE values indicate better predictive performance and less variability in measurements. In this analysis, MSE values are generally higher than MAE values, indicating more significant errors in selected measurement values. Like MAE, RF muscles exhibit lower MSE values than VM muscles, indicating better agreement between measurements. When measurements from both muscles are combined, the MSE values follow a similar trend, with the lowest MSE observed in Stiffness (0.0251). Moreover, it could be seen that for stiffness, the differences between measurers were visibly low, indicating a high correlation between the measurements. It should also be noted that while measurements for both muscles were analyzed instead of only one selected muscle, the correlation between measurements was higher than for the VM case, with lower differences represented as MAE and MSE. For the MAE measure, a lower value indicates better agreement between measurements. In this analysis, RF muscles generally exhibit lower MAE values than VM muscles, suggesting higher consistency in measurements for this muscle within the analyzed specialists. To evaluate the influence of individual measures on the correlation of results, the data from the different groups were integrated into one collective vector of values. This approach enabled a holistic examination of the correlations between measurements, focusing primarily on disparities attributable to the measurer's expertise or technique. The reliability coefficients, along with their 95% confidence intervals, provided valuable insights into the consistency and agreement among raters in assessing various muscle characteristics for the evaluated muscles (Table 2).

**Table 2:** Comparative analysis of correlation measures (Pearson, mean absolute error (MAE), mean absolute error divided by the average value (MAE%), and mean squared error (MSE) between examined cases of muscle parameters measurements (RF, rectus femoris; VM, vastus medialis, both) regarding three characteristics of muscle tone, elasticity, and stiffness.

Metric	Muscle tone [Hz]			Elasticity [arb]			Stiffness [N/m]		
	RF	VM	Both	RF	VM	Both	RF	VM	Both
Pearson	0.8164	0.8298	0.8726	0.8298	0.8369	0.8871	0.8876	0.7253	0.8208
MAE	0.5191	9.9148	0.7414	9.9148	23.1064	16.5106	0.0992	0.1341	0.1167
MAE%	3.4	6.3	4.5	3.6	8.5	5.5	6.4	8.7	7.4
MSE	0.5855	2.2594	1.4224	261.0638	966.2553	613.6595	0.0181	0.0322	0.0251

**Figure 3:**
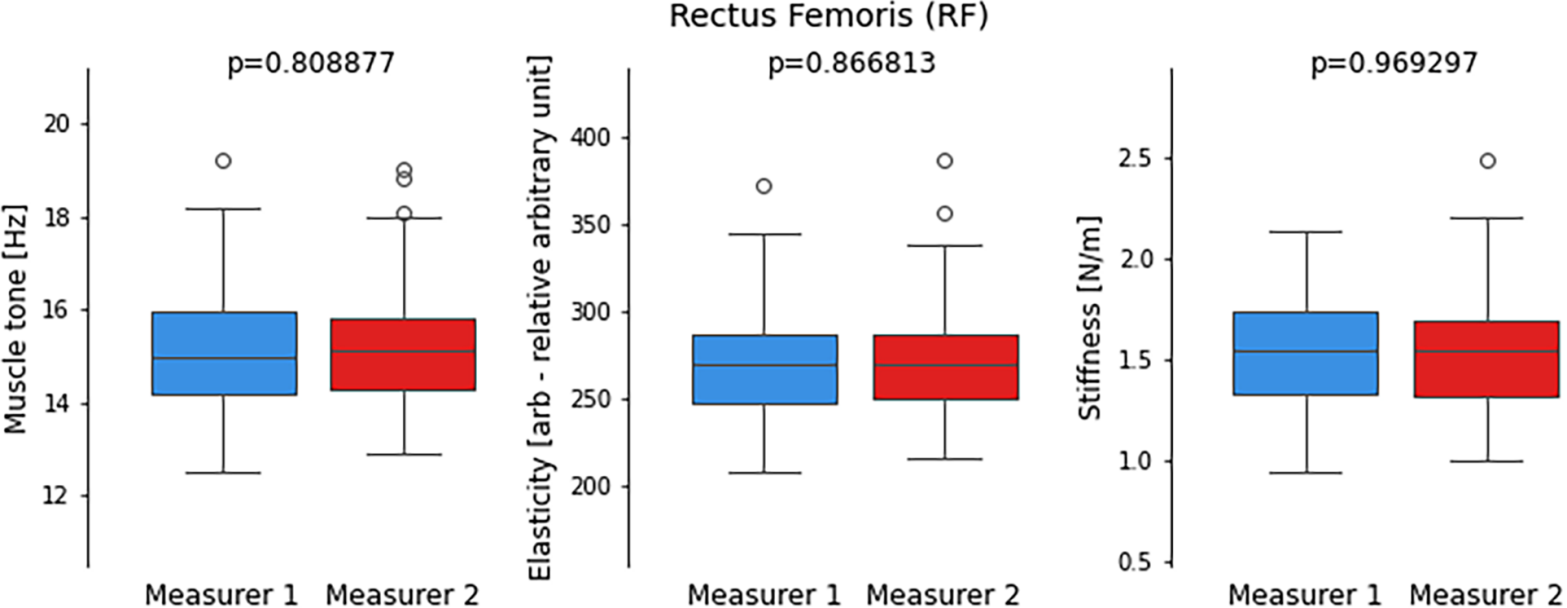
Comparison of distribution of values from performed measurements by two measurers for the Rectus femoris (RF) including muscle tone, elasticity, and stiffness parameters. Each boxplot represents the distribution of measurements by two different measurers, with whiskers indicating the range of non-outlier data. Outlying data points, represented by dots, are also depicted on the plot to visualize any extreme values. The statistical significance of the observed differences between the two sets of measurements is determined by the *p*-value obtained from the *t*-student test.

In the analysed parameters of muscle tone, elasticity and stiffness of the RF there were no significant statistical differences between the two measurers. There were outliers recorded in every examined parameter (Figure 3).

**Figure 4:**
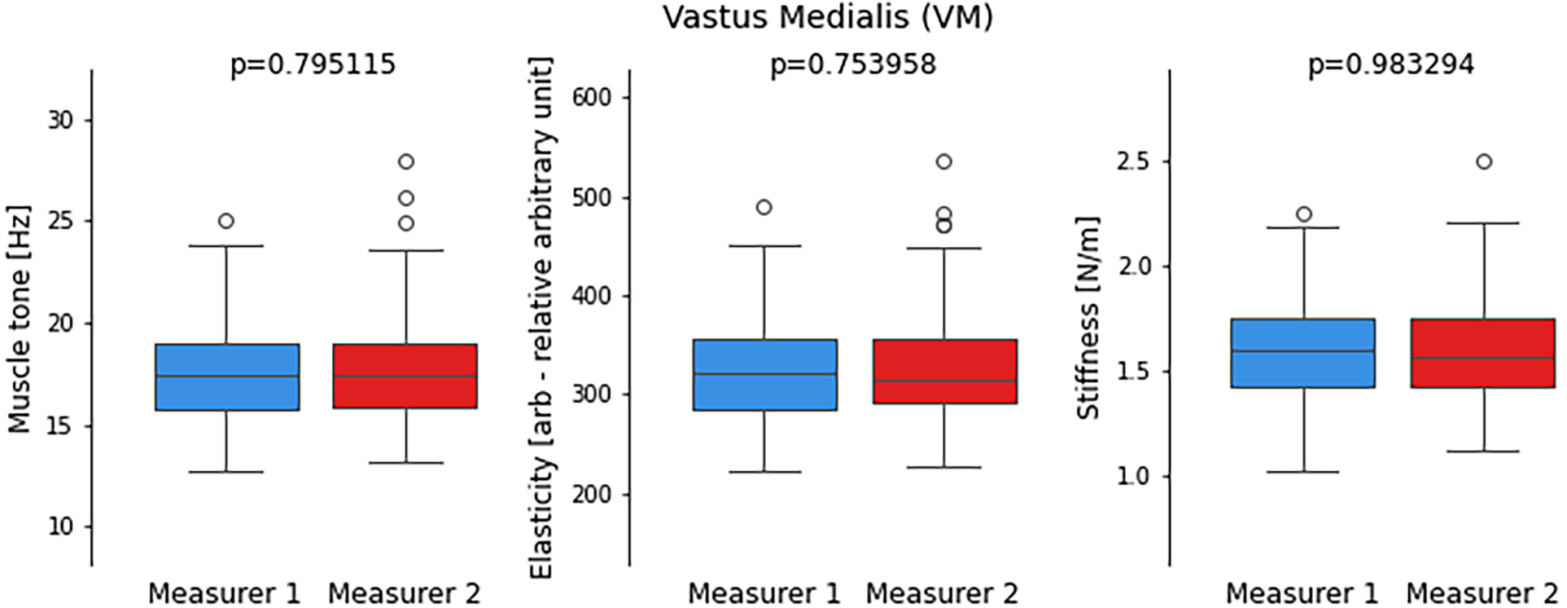
Comparison of distribution of values from performed measurements by two measurers for the Vastus Medialis (VM) including muscle tone, elasticity, and stiffness parameters. Each boxplot represents the distribution of measurements by two different measurers, with whiskers indicating the range of non-outlier data. Outlying data points, represented by dots, are also depicted on the plot to visualize any extreme values. The statistical significance of the observed differences between the two sets of measurements is determined by the *p*-value obtained from the t-student test.

Similarly to the above, in the analysed parameters of muscle tone, elasticity and stiffness of the VM there were no significan statistical differences between the two measurers. There were outliers recorded in every examined parameter (Figure 4).

**Figure 5:**
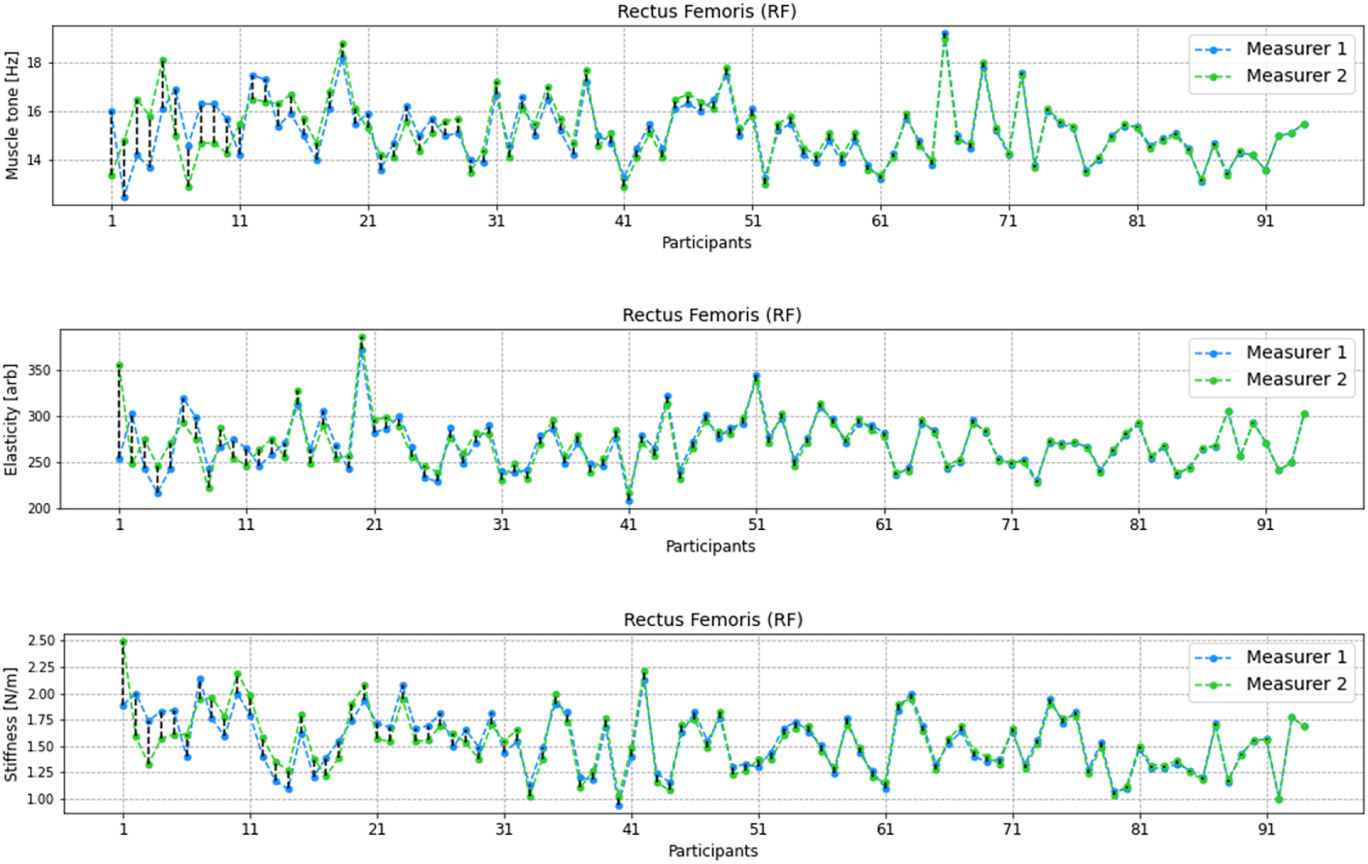
Differences between the measurers 1 and 2 for every participant in adequate units for muscle tone, elasticity and stiffness for RF muscle. The results were sorted by differences between measurer 1 and 2 from the highest to the lowest.

**Figure 6:**
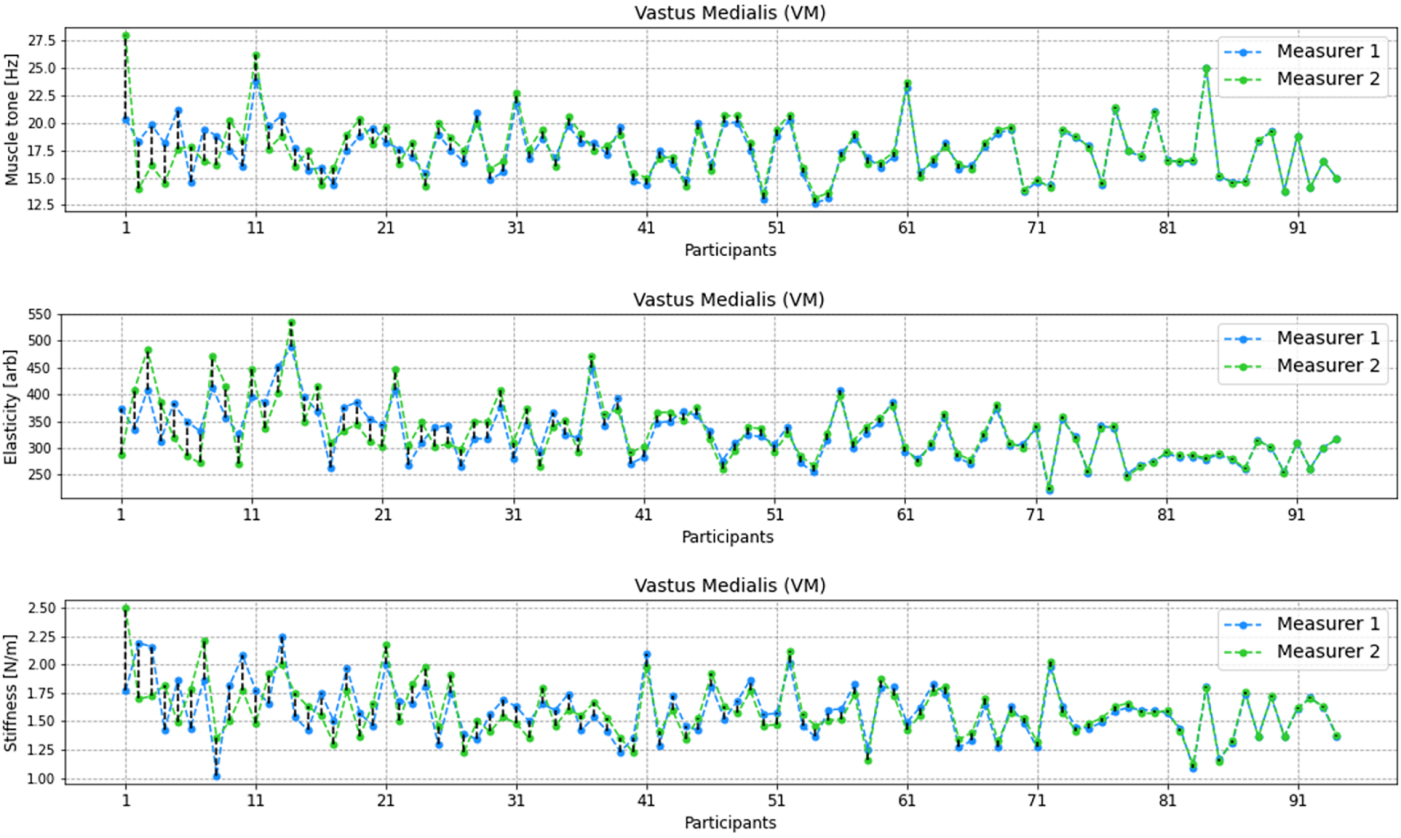
Differences between the measurers 1 and 2 for every participant in adequate units for muscle tone, elasticity and stiffness for VM muscle. The results were sorted by differences between measurer 1 and 2 from the highest to the lowest.

Figures 5, 6 shows the differences in results performed by the two measurers for RF and VM muscles. The lowest differences were observed for elasticity of RT and the highest differences were obserwed for muscle tone of RF muscle.

### 3.3 Interclass correlation results

**Figure 7:**
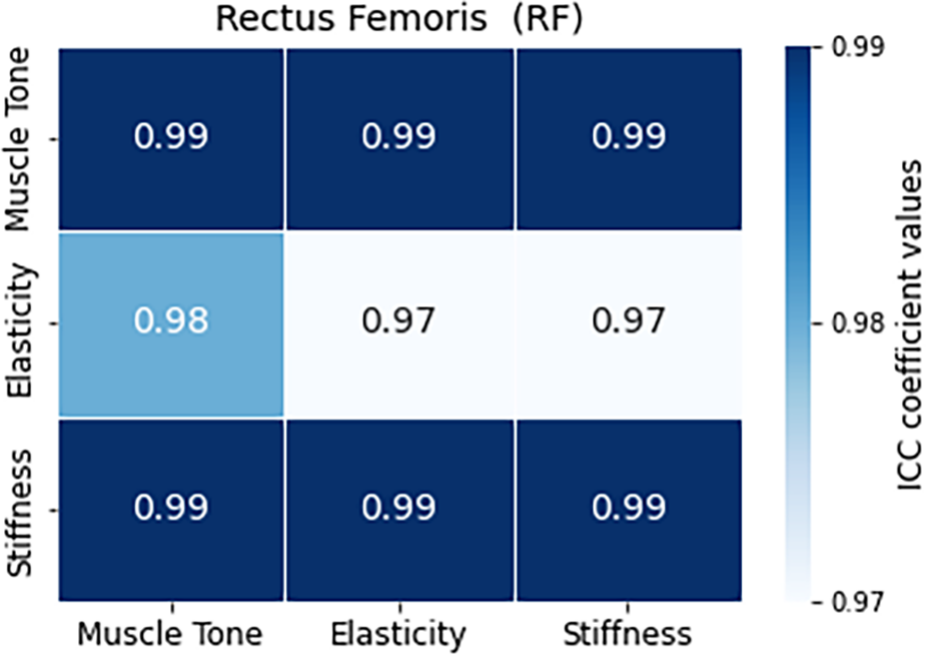
Comparative analysis of intraclass correlation (ICC) values for RF muscle and the evaluated characteristics of tension, elasticity, and stiffness.

**Figure 8:**
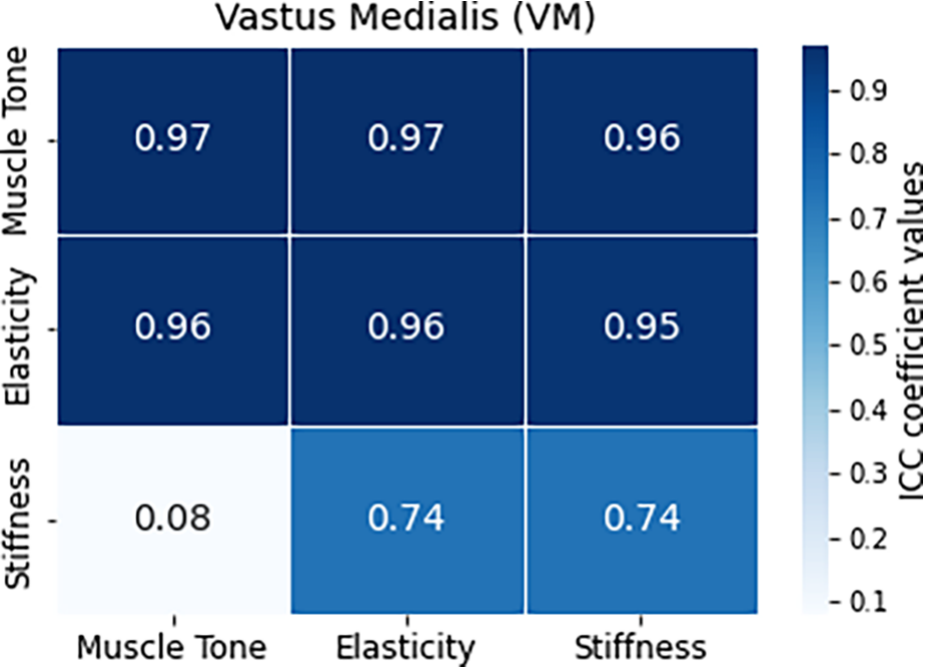
Comparative analysis of intraclass correlation (ICC) values for VM muscle and the evaluated characteristics of tension, elasticity, and stiffness.

All three ICC values (ICC_1,2_, ICC_2,2_, and ICC_3,1_) are very high, indicating high reliability in assessing tension in the RF muscle (Figure 7). The 95% confidence intervals are narrow, meaning high precision in estimating the true ICC values for tension, elasticity and stiffness characteristics. The widest range of values could be observed for the elasticity parameter for ICC_3,2_ metric. The ICC values for tension and elasticity in VM are high, indicating good reliability, with slightly lower values than in the case of RF (Figure 8). The confidence intervals are wider, suggesting some variability in the estimates. Interestingly, the ICC values for stiffness and ICC_1,2_ metric in VM indicates poor reliability with a near-zero ICC value and a wide confidence interval crossing zero, suggesting low agreement among raters. However, both ICC_2,2_ and ICC_3,2_ show a significantly higher correlation in ICC values, indicating better agreement among raters in assessing stiffness. The confidence intervals for both models are relatively wide, indicating variability in the estimates.

### 3.4 Correlation results of muscles biomechanical parameters between the examined groups

To this end, measurements for each group were treated separately to analyze whether the differences between groups were significant. Moreover, including these varied groups facilitated a comprehensive assessment of muscle characteristics across different athletic disciplines and general demographics. The number of individuals in each group was different. To this end, to count the differences between the values derived from the measurements for the individual groups, a procedure was used in which the mean value was initially calculated from the entire data vector, including the measurements for a given muscle and a given aspect by both measuring persons. This average was calculated for a single test group. Then, the mean value for the next test group was calculated using the same process. The two average values obtained were then used to calculate the absolute value from the difference of these averages. This result was then divided by the average of these values, indicating the percentage differences between the groups for the muscles in question and their test properties.

A systematic approach was implemented to quantify differences in measurement values across individual groups to account for variations in group sizes. The presented matrices compare different sports groups based on the RF muscle measurements: muscle tone, elasticity, and stiffness (Table 3). For tension, footballers and volleyballers exhibit relatively low differences (0.00645), indicating similar tension levels between these two groups. Handballers also show minimal differences with footballers (0.02126) and volleyballers (0.02770), suggesting comparable tension levels. MMA fighters demonstrate slightly higher differences with footballers (0.03454) and volleyballers (0.04098) but still relatively close tension levels. The untrained group displays similar tension levels with MMA fighters (0.00139) but slightly higher than other sports groups. However, these values oscillate between 0.1% to 4%, showing high similarity in the analyzed aspect.

**Table 3:** Comparative analysis of the correlation between examined groups regarding the RF muscle and tension, elasticity and stiffness parameters calculated as the difference between the average value of tension of the RF muscle of two groups divided by the mean of those averages.

	Group	Footballers	Volleyballers	Handballers	MMA fighters	Untrained
Muscle tone	Footballers	0.00000	0.00644	0.02125	0.03453	0.03315
	Volleyballers		0.00000	0.02770	0.04097	0.03959
	Handballers			0.00000	0.01329	0.01190
	MMA fighters				0.00000	0.00138
	Untrained					0.00000
Elasticity	Footballers	0.00000	0.01985	0.02452	0.05370	0.04578
	Volleyballers		0.00000	0.04435	0.07345	0.06558
	Handballers			0.00000	0.02918	0.02128
	MMA fighters				0.00000	0.00790
	Untrained					0.00000
Stiffness	Footballers	0.00000	0.06104	0.01470	0.05686	0.01015
	Volleyballers		0.00000	0.04637	0.00419	0.05092
	Handballers			0.00000	0.04218	0.00455
	MMA fighters				0.00000	0.04673
	Untrained					0.00000

For elasticity, similar to tension, footballers, and volleyballers show low differences (0.01986), indicating comparable elasticity levels. Handballers exhibit slightly higher differences with footballers (0.02452) and volleyballers (0.04436), showing some variation in elasticity. MMA fighters demonstrate slightly higher differences with footballers (0.05367) and volleyballers (0.07345), indicating distinct elasticity levels. The untrained group displays intermediate differences with other sports groups in elasticity measurements. The obtained differences varied in the range from 0.7% to 7.3%. The highest difference was obtained between MMA fighters and footballers in terms of the elasticity of RF.

Footballers and volleyballers show relatively low differences (0.06104) for stiffness, indicating similar stiffness levels. Handballers exhibit slightly higher differences with footballers (0.01471) and volleyballers (0.04638), suggesting some variation in stiffness. MMA fighters demonstrate noticeable differences with footballers (0.05686) and volleyballers (0.00420), indicating distinct stiffness levels. The untrained group displays intermediate differences with other sports groups in stiffness measurements. The obtained values ranged from 0.4% to 5.6%, being slightly more spread than in the case of tension but less spread than in the case of elasticity.

The matrices provided below compare different sports groups based on measurements of the VM muscle in the same three characteristics: muscle tone, elasticity, and stiffness (Table 4). For tension, footballers and handballers display relatively low differences (0.04119), indicating similar tension levels, while volleyballers show slightly higher differences with footballers (0.04276) and handballers (0.08380), suggesting some variation in tension. MMA fighters demonstrate notably higher differences with footballers (0.10635) and handballers (0.14843), indicating distinct tension levels. The untrained group's results were similar to those of handballers, while the highest discrepancies were observed compared to those of volleyballers. Thus, comparing MMA fighters to footballers and handballers and the untrained group to volleyballers shows significant differences in those groups regarding the tension of VM muscle.

**Table 4:** Comparative analysis of the correlation between examined groups regarding the VM muscle and muscle tone, elasticity and stiffness parameters was calculated as the difference between the average value of tension of the VM muscle of two groups divided by the mean of those averages.

	Group	Footballers	Volleyballers	Handballers	MMA fighters	Untrained
Muscle tone	Footballers	0.00000	0.04275	0.04118	0.10634	0.06257
	Volleyballers		0.00000	0.08379	0.14842	0.10505
	Handballers			0.00000	0.06544	0.02144
	MMA fighters				0.00000	0.04406
	Untrained					0.00000
Elasticity	Footballers	0.00000	0.05298	0.02568	0.09863	0.06371
	Volleyballers		0.00000	0.07856	0.15083	0.11631
	Handballers			0.00000	0.07314	0.03809
	MMA fighters				0.00000	0.03514
	Untrained					0.00000
Stiffness	Footballers	0.00000	0.00854	0.02096	0.00810	0.00316
	Volleyballers		0.00000	0.01242	0.00044	0.01170
	Handballers			0.00000	0.01286	0.02412
	MMA fighters				0.00000	0.01126
	Untrained					0.00000

For elasticity, footballers and handballers show relatively low differences (0.02568), indicating comparable elasticity levels. Volleyballers exhibit slightly higher differences with footballers (0.05299) and handballers (0.07857), suggesting some variation in the examined aspect. MMA fighters demonstrate noticeable differences with footballers (0.09864) and handballers (0.15084), indicating distinct elasticity levels. The untrained group's results were similar to MMA fighters and the least similar to the volleyballers group.

For stiffness, footballers and MMA fighters show relatively low differences (0.00855), indicating similar stiffness levels. Volleyballers exhibit slightly higher differences with footballers (0.00855) and MMA fighters (0.01242), suggesting some variation in stiffness. Handballers demonstrate similar differences with footballers (0.02097) and MMA fighters (0.01287). The untrained group was highly similar to other evaluated groups of athletes.

## 4 Discussion

Our study aimed to determine the usefulness and repeatability of MyotonPro. The results of our study showed that the biomechanical properties of RF and VM muscles, such as elasticity, stiffness, and muscle tension, as measured by MyotonPro, show uniformity between sites and researchers performing the measurements. Analysis of the results between groups shows that for some sports groups, measurements for RF and VM muscles show slight differences in individual groups, especially in muscle stiffness, which may result from other specific requirements and training loads. Our study shows that while certain sports groups share similarities in muscle measurements, there are few visible differences across selected groups, particularly in elasticity and stiffness, which may reflect the specific demands and training regimens.

An essential factor in the development of sports science is constant work on standardizing measurement systems and assessing the reliability of measuring instruments (9, 20). Since many factors can influence the measurement of biomechanical changes in muscles (11), research should be conducted to identify these conditions to minimize the impact of any stimuli interfering with the measurements and strictly follow the principles of proper experimentation. The proper selection of measuring equipment (25, 54) and the measurement itself are crucial at this stage (16). Therefore, it must be performed according to specific standards, which in myotonometry still need ready protocols (14). Standardization using MyotonPro enables comparison of test results and determines their correct interpretation (1). Failure to follow methodological guidelines in research often leads to measurement errors, which results in misleading conclusions (17).

The biomechanical properties of the quadriceps femoris muscle are influenced by many factors, which can be divided into several main categories: genetic, biomechanical, training, and lifestyle and health (34). Genetic elements can be divided into electrical switches and their stiffness. For example, some people may have a genetic component (55, 56). The scientific literature found that older athletes may experience greater muscle stiffness in certain areas (57). Congenital body asymmetries influence the stiffness variability (21).

Muscle structure and tissue elasticity differences between men and women can affect muscle stiffness (18). Differences in exercise and sports movement techniques and load volumes can affect the load and tension of different muscle groups, leading to variable stiffness and remodeling of these muscles (3). In our subjects’ case, the quadriceps muscles that put the most strain on them were volleyball players and soccer players. Asymmetries and disproportions in muscle strength and flexibility can lead to uneven loading on muscles and joints, which can cause variable stiffness (21).

Adequate hydration and a diet rich in essential nutrients are vital to maintaining muscle elasticity and preventing muscle stiffness (58). Finally, another important factor is the athlete's recovery; adequate sleep and recovery time are essential for proper muscle function and preventing excessive stiffness (5, 32).

There is evidence that the psychological profile, reactions to stress and mental problems can influence muscle tone disorders and lead to increased stiffness and pain (59, 60).

In addition, past injuries and contusions may lead to permanent muscle stiffness due to tissue scarring and changes in muscle structure (61), and some chronic, autoimmune, or neurological diseases may affect muscle tension and elasticity Mckeon and Vincent (62).

The literature describes MyotonPRO as a reliable device for assessing the mechanical properties of several muscles and tendons (26). However, studies published in the literature have yet to use MyotonPRO to measure the reliability of RF and VM for many groups practicing sports and healthy volunteers who do not. The results for the VM muscle show some significant differences between selected groups and evaluated characteristics. The most varied groups for elasticity were MMA fighters and handballers, with a 15% difference in the results. On the other hand, footballers and volleyballers were highly similar in terms of the analyzed aspects of VM characteristics.

It is worth noting that we observed significant differences in these parameters among non-athletes. This highlights the potential impact of regular training and repeated exercise on the uniformity of muscle tension and stiffness within a sport (34). This is an essential tip for coaches and future researchers, which may allow the so-called optimal muscle stiffness profile in a given sports discipline to be studied in the future. The lower stiffness value may be related to race, gender, research, or training (42). Our results show that athletes adhere to the highest values compared to untrained people.

Optimizing muscle tension and flexibility in people performing various forms of physical activity as amateurs does not affect their ability to generate muscle power (63). Although it may not significantly impact the result, it has a confirmed impact on the risk of injury (64). The situation is entirely different in the case of athletes, where the biomechanical parameters of the quadriceps muscles influence the result (65). Therefore, our results in this area may help plan monitoring of fatigue after physical exercise by observing changes.

Our observations of heightened muscle stiffness in MMA fighters, compared to other groups and within the MMA fighters, could have significant implications for training methods in this sport. This unique condition may result from the specific movements and isometric work required in MMA, particularly by dominant strikers (66). It is worth noting that MMA training units incorporate muscle strength training programs and heavy athletics methods (67). Some studies suggest that changes in quadriceps muscle stiffness and tension are variable and depend on the measurement site. Still, these differences do not affect the overall function of the quadriceps muscles (36). It is suggested that greater tension in these muscles occurs in athletes who neglect stretching during training (68). In our research, MMA fighters demonstrate the highest degree of flexibility of the quadriceps muscles due to the specificity of training and the need to perform high kicks (69). Other studies suggest that relative quadriceps EMG activity was higher across different activities/exercise modes in older adults compared to younger adults, which may be related to various metabolic responses of the neuromuscular system (70).

Increased stiffness appears to benefit athletic performance, so practitioners working with athletes required to perform dynamic activities may want to consider the impact of stiffness on athletic performance (36). Scientific literature on the biomechanics of the quadriceps femoris muscle allows for separating this muscle into one functional structure (22). However, some studies suggest that the induction of femoral cartilage stress is more advanced in the case of changes in muscle forces for the medial head of the quadriceps femoris (71). Volleyball players who showed high values of VM stress in our tests, taking into account significant eccetric loads on this muscle, should pay attention to avoiding these stresses to prevent cartilage damage.

There is a balance between muscle strength and muscle stiffness, which allows the storage and release of elastic energy and facilitates the working conditions of muscle fibers (54). Athletes have greater muscle strength and tendon stiffness, which are used to achieve athletic performance (72). Our research presented results suggesting greater resting stiffness in trained people than in untrained people.

MyotonPro measurements do not require the active participation of the athlete or patient. The results presented in the scientific literature correlating muscle strength with stiffness, flexibility, and muscle tension may have potential practical applications for any professional who wants to estimate the strength of the quadriceps femoris muscle without active effort on the part of the subject (73).

In summary, the reliability coefficients suggest high reliability in assessing muscle characteristics, particularly tension and elasticity, for both RF and VM muscles. Stiffness assessment initially showed poor reliability in VM but improved significantly in subsequent models. These results provide confidence in the consistency and agreement among raters in evaluating muscle characteristics, essential for accurate clinical assessments and interventions (Table 3).

## 5 Limitations of the study

Although myotonometry appears to be a simple and reliable measurement method, two critical issues must be considered. First, more types of myotones are still needed for reliability analysis, and their inter-rater reliability needs to be assessed. Additional limitations of these studies are that long-term reproducibility of the results was not performed, and a validation study, such as ultrasonic elastography, was necessary. In further studies, the efficacy and reliability could be tested in more locations on other muscles, including antagonists. Variables required during evaluation would be the type of side, presence of pain, another stereogenic factor or anxiety, temperature changes, noise and evaluation of different muscle points located in the muscle belly and tendon as well as measurements of the influence of body fat. What is more—future studies should include females and different age groups—young athlethes and untrained individuals as well as elderly people.

## 6 Conclusions

Myotometry seems to be an essential and reliable complementary tool in assessing the viscoelastic properties of the quadriceps femoris muscles in people with different levels of motor preparation. Overall, the results suggest that although some sport groups perform similarly on muscle measures, there are some apparent differences between the selected groups, particularly in flexibility and stiffness, which may reflect the specific demands and training regimens associated with each sport.

## 7 Practical application

Myotonometry is a reliable and well validated tool to measure muscle biomechanical properities and can be used both in athlethes and non training individuals. The measurements in ideal situation should be done by the same practicioner every time, thus, if necessary can be conducted by another investigator without raising doubts according the reliability of the results. Myotonometry is a valuable tool in practice and scientific research in examining the influence of exercise, recovery and other interventions on biomechanical parameters of the muscles.

## Data Availability

The datasets presented in this study can be found in online repositories. The names of the repository/repositories and accession number(s) can be found in the article/Supplementary Material.
